# Fibrinogen and Atherosclerosis

**DOI:** 10.5935/abc.20170017

**Published:** 2017-02

**Authors:** Levent Cerit

**Affiliations:** Near East University - Nicosia - Chipre

**Keywords:** Fibrinogen, Coronary Artery Disease, Obesity, Dyslipidemias

**To the Editor,**

I have read with great interest the article entitled "Early Markers of Atherosclerotic Disease in Individuals with Excess Weight and Dyslipidemia" by Menti et al.^1^ recently published in *Arquivos Brasileiros de Cardiologia* 2016; 106: 457-63. The investigators reported that fibrinogen is associated with subclinical atherosclerosis in individuals with excess weight.^1^

Several studies have shown that high serum levels of fibrinogen are strongly associated with coronary artery disease.^2,3^ High serum levels of fibrinogen may contribute to vascular disease by increasing blood viscosity, stimulating fibrin formation, or increasing platelet-platelet interaction.^2,3^

Catena et al.^4^ reported that plasma homocysteine (Hcy) levels were directly correlated with age, waist circumference, fasting glucose, triglyceride, uric acid, and fibrinogen levels, and inversely correlated with creatinine clearance and high-density lipoprotein cholesterol, vitamin B12, and folate levels. Low vitamin B12 concentration and hyperhomocysteinemia are common, and might affect serum fibrinogen levels.

25-hydroxyvitamin D [25(OH)D] deficiency with increased risks of cardiovascular disease and venous thromboembolism may relate to adverse hemostatic and inflammatory responses. Blondon et al.^5^ reported that low levels of serum [25(OH)D] were cross-sectionally associated with higher levels of interleukin-6, homocysteine, total tissue factor pathway inhibitor and plasminogen activator inhibitor-1.

In light of these findings, it might be beneficial to evaluate serum levels of vitamin B12, Hcy, and [25(OH)D] because of their close association with fibrinogen levels.

## References

[1] Menti E, Zaffari D, Galarraga T, Lessa JR, Pontin B, Pellanda LC (2016). Early markers of atherosclerotic disease in individuals with
excess weight and dyslipidemia. Arq Bras Cardiol.

[2] Folsom AR, Wu KK, Rosamond WD, Sharrett AR, Chambless LE (1997). Prospective study of hemostatic factors and incidence of coronary
heart disease: the Atherosclerosis Risk in Communities (ARIC)
Study. Circulation.

[3] Heinrich J, Balleisen L, Schulte H, Assmann G, Loo J (1994). Fibrinogen and factor VII in the prediction of coronary risk:
results from the PROCAM study in healthy men. Arterioscler Thromb.

[4] Catena C, Colussi G, Nait F, Capobianco F, Sechi LA (2015). Elevated homocysteine levels are associated with the metabolic
syndrome and cardiovascular events in hypertensive patients. Am J Hypertens.

[5] Blondon M, Cushman M, Jenny N, Michos ED, Smith NL, Kestenbaum B, de Boer IH (2016). Associations of serum 25-hydroxyvitamin D with hemostatic and
inflammatory biomarkers in the multi-ethnic study of
atherosclerosis. J Clin Endocrinol Metab.

