# Supplementary material for: Fibrinogen and Atherosclerosis
Source: Arq Bras Cardiol. 2017 Feb;108(2):189–90. doi: 10.5935/abc.20170017 (PMC5344667; doi:10.5935/abc.20170017)
Supplement: Related texts

# Reply

**Authors:** Eduardo Menti

We were honored by your interest in our article entitled *"Early Markers of Atherosclerotic Disease in Individuals with Excess Weight and Dyslipidemia"* published in *Arquivos Brasileiros de Cardiologia.*

The finding of high fibrinogen levels in patients with atherosclerotic disease has generated a large volume of clinical evidences in the past few decades, and has been considered a risk marker for cardiovascular events. High fibrinogen levels are also known to promote atherosclerotic disease by increasing blood viscosity, stimulating fibrin formation and increasing platelet aggregation. In the setting of an inflammatory status such as that seen in individuals with excess weight, the higher hepatic production of fibrinogen regulated by inflammatory cytokines may be an important link in the progression of the atherosclerotic disease in its different subclinical and clinical stages. Our study had a small sample size, however large enough to bring this association to light.^1^

As regards the assessment of serum levels of vitamin B12 and vitamin D, in a recent study with a small sample, Baser et al^2^ observed the association of vitamin D deficiency with high fibrinogen levels and pro-oxidative serum markers. Vitamin B12 deficiency, by hyperhomocysteinemia induction, also plays a role in the development of cardiovascular disease. Every 5-mcmol/L increase above 10 mcmol/L in serum levels of homocystein is associated with a 20% increase in the risks of circulatory disorders.^3^

Although the assessment of changes in endothelial function in patients with vitamin B12 and vitamin D deficiency had not been included in this study, it is a promising research field. In a study with a small sample assessing endothelial function using flow-mediated brachial artery dilatation in patients with vitamin B12 deficiency, increased dilatation after proper vitamin B12 replacement was observed.^4^ A similar finding was observed in a sample of individuals undergoing hemodialysis after vitamin D replacement.^5^

We thank Dr. Cerit's remarks and are satisfied to stimulate discussion regarding this important field of clinical cardiology, which has been a frequent concern in daily practice, in view of the growing incidence of obesity among our society.

**Eduardo Menti**

## References

[1] Menti E, Zaffari D, Galarraga T, Lessa JR, Pontin B, Pellanda LC (2016). Early markers of atherosclerotic disease in individuals with
excess weight and dyslipidemia. Arq Bras Cardiol.

[2] Baser H, Can U, Baser S, Hidayetoglu BT, Aslan U, Buyuktorun I (2015). Serum total oxidant/anti-oxidant status, ischemia-modified
albumin and oxidizedlow density lipoprotein levels in patients with
vitamin D deficiency. Arch Endocrinol Metab.

[3] Pawlak R (2015). Is vitamin B12 deficiency a risk factor for cardiovascular
disease in vegetarians?. Am J Prev Med.

[4] Kwok T, Qiao M, Tam L, Poon YK, Ahuja AT (2012). Vitamin B-12 supplementation improves arterial function in
vegetarians with subnormal vitamin B-12 status. J Nutr Health Aging.

[5] Karakas Y, Sahin G, Urfali FE, Bal C, Degirmenci NA, Sirmagul B (2016). Effect of vitamin D supplementation on endothelial
dysfunction in hemodialysis patients. Hemodial Int.
